# Magnetic resonance imaging and computed tomography of equine cheek teeth and adjacent structures: comparative study of image quality in horses in vivo, post-mortem and frozen-thawed

**DOI:** 10.1186/s13028-019-0495-8

**Published:** 2019-12-10

**Authors:** Christin Röttiger, Maren Hellige, Bernhard Ohnesorge, Astrid Bienert-Zeit

**Affiliations:** Clinic for Horses, University of Veterinary Medicine Hannover, Foundation, Bünteweg 9, 30559 Hannover, Germany

**Keywords:** Cheek teeth, CT, Equine dental imaging, High-field magnetic resonance imaging, Horse, Scoring system, Periodontal ligament

## Abstract

**Background:**

The use of cadavers for radiology research methodologies involving subjective image quality evaluation of anatomical criteria is well-documented. The purpose of this method comparison study was to evaluate the image quality of dental and adjacent structures in computed tomography (CT) and high-field (3 T) magnetic resonance (MR) images in cadaveric heads, based on an objective four-point rating scale. Whilst CT is a well-established technique, MR imaging (MRI) is rarely used for equine dental diagnostics. The use of a grading system in this study allowed an objective assessment of CT and MRI advantages in portraying equine cheek teeth. As imaging is commonly performed with cadaveric or frozen and thawed heads for dental research investigations, the second objective was to quantify the impact of the specimens’ conditions (in vivo, post-mortem, frozen-thawed) on the image quality in CT and MRI.

**Results:**

The CT and MR images of nine horses, focused on the maxillary premolar 08s and molar 09s, were acquired post-mortem (Group A). Three observers scored the dental and adjacent tissues. Results showed that MR sequences gave an excellent depiction of endo- and periodontal structures, whereas CT produced high-quality images of the hard tooth and bony tissues. Additional CT and MRI was performed in vivo (Group B) and frozen-thawed (Group C) in three of these nine horses to specify the condition of the best specimens for further research. Assessing the impact of the specimens’ conditions on image quality, specific soft tissues of the maxillary 08s and 09s including adjacent structures (pulps, mucosa of the maxillary sinuses, periodontal ligament, soft tissue inside the infraorbital canal) were graded in group B and C and analysed for significant differences within CT and MR modalities in comparison to group A. Results showed that MRI scores in vivo were superior to the post-mortem and frozen-thawed condition.

**Conclusions:**

On comparing the imaging performance of CT and MRI, both techniques show a huge potential for application in equine dentistry. Further studies are needed to assess the clinical suitability of MRI. For further research investigations it must be considered, that the best MR image quality is provided in live horses.

## Background

The imaging of equine cheek teeth pathologies, such as apical periodontitis [1], pulpitis [2], infundibular caries [3] or ascending infections [4], has been expanded substantially. Although the clinical dental examination is always the basic start, supplementary imaging might be necessary to make a diagnosis [1]. Therefore, knowledge of the physiological depiction of dental, periodontal and adjacent structures in different imaging modalities is essential to obtain accurate diagnoses.

Radiography has always been the primary, established and most widely used standard for dental imaging in horses when comparing different imaging modalities [5]. Diagnostic imaging procedures added more recently, such as computed tomography (CT) and magnetic resonance imaging (MRI), are characterized by high tissue contrast and the possibility of multiplanar or three-dimensional reconstructions without superimposition [6, 7]. Whereas CT has already been established for diagnosing equine dental pathologies [3, 8, 9], the possibilities of dental MRI diagnoses are rarely used in equine dentistry. The MRI has the potential to produce images with excellent detail of dental soft tissues [2, 10]. Regarding clinical patients, MRI might help to assess the vitality of pulp tissue. The study of [2] showed that evaluation of dental pulp in equine cheek teeth is possible using MRI, as pulp with a blurred or enlarged MR signal was considered diseased. With the information regarding which pulp horn is vital or necrotic, endodontic treatment might be more accurate and purposeful. If it remains unclear (after clinical, radiographic and CT examination) whether the periodontal ligament (PDL) is involved in the pathological dental progress, MRI might help to evaluate the vitality of the PDL due to the different intensities depicted in MRI [11]. Endodontic treatment [12] or replantation [13, 14] of apically infected cheek teeth might be a promising alternative to conventional tooth extractions in teeth with a vital PDL. Recent studies compared CT and 3.0 T dental MRI quantitatively in horses, aiming to highlight the best imaging technique for each structure [10]. Qualitative head-to-head comparisons of CT and different MRI protocols, based on a scoring system, have already been performed in human medicine [15]. The general differences between CT and MRI in dental imaging are widely reported in equine medicine, but a rating scale for detailed, more objective results has not yet been used.

Many research investigations have been conducted as there is a need for a better understanding of the pathogenesis of dental diseases. Most of these research examinations have been performed with cadaveric heads and some imaging procedures are performed on frozen and thawed heads. A decrease of the magnetic resonance (MR) signal was described for equine limbs when evaluating defined structures immediately post-mortem and frozen-thawed [16]. Regarding equine dental imaging, there is currently a lack of information about whether the image quality suffers in equine heads post-mortem or frozen-thawed.

The purpose of the current study was to evaluate the general image quality and visibility of dental, periodontal and adjacent structures in CT and different high-field MRI sequences based on a four-point grading scale in cadaveric heads. Another aim was to assess the impact of the condition of the specimens (horses alive, heads post-mortem or frozen-thawed) on the CT and MRI quality and detailed representation of the structures mentioned above. The authors hypothesize that the image quality may achieve the same results in CT images in all groups, but MRI scores may achieve better results regarding the image quality for the dental and periodontal tissues in horses alive compared to those post-mortem or frozen-thawed.

## Methods

### Specimens and study design

**Fig. 1:**
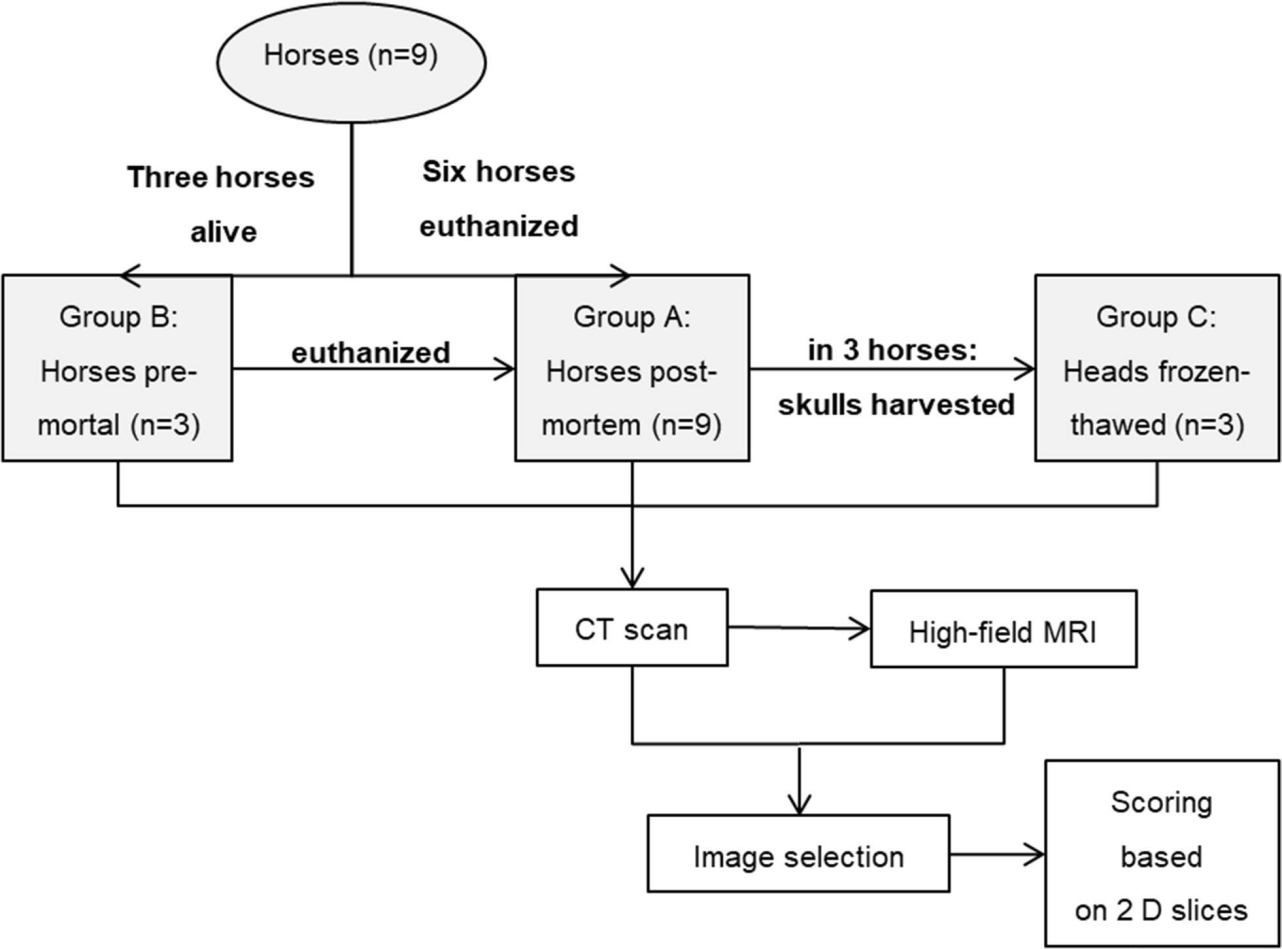
Flow chart illustrating the study design. *CT* computed tomography, *MRI* magnetic resonance imaging. *Horses of group B and C were the same

Nine Warmblood horses were prospectively chosen to undergo CT and high-field MRI to display selected maxillary cheek teeth, their periodontal tissues and adjacent structures. Figure 1 illustrates how the method comparison study was conducted. All horses examined post-mortem (group A, n = 9) underwent a CT and MRI acquisition within four hours after euthanasia. The population of group A consisted of five mares and four geldings with a median age of 8.2 years (2.3 to 22.1 years). All horses were owned by the clinic (University of Veterinary Medicine Hannover, Clinic for Horses, Germany) and humanely put down for reasons unrelated to the study. One of the authors (ABZ) decided on the inclusion of each subject: none of the horses had a known history or clinical signs of a paranasal sinus or dental disease. Any clinical signs of dental (e.g. abnormal feed intake or quidding) or sinus disease (e.g. nasal discharge) resulted in exclusion.

The current examinations were linked to another research study. All nine horses examined post-mortem were derived from the other scientific survey. A part of the study population (group B) has also been examined in vivo. The CT and MR examinations in vivo were only possible for three of the nine horses in the present trial due to the experimental set-up of the linked study. The median age of this study population (n = 3) was 9.1 years. The horses of group B were euthanized within 14 to 16 days after CT and MRI acquisition under general anaesthesia. The heads of these horses were harvested post-mortem at the atlanto-occipital joint and frozen (− 20 °C) for 2 weeks (group C, n = 3). The CT and MRI datasets were acquired a third time within 48 h after the heads were thawed. The heads were warmed up to a core temperature of 15 °C to prevent imaging artefacts that might occur due to frozen tissues. When assessing the CT and MR images, the modified Triadan system was used for numbering the maxillary cheek teeth [17]. A total of 36 cheek teeth were examined. The images evaluated included 18 maxillary Triadan 08s and 18 maxillary Triadan 09s.

### Imaging

The examinations were acquired at the University of Veterinary Medicine Hannover, Foundation. All groups were examined with the same imaging settings for the CT and MRI acquisition. The horses or heads were first positioned on a stationary CT table in right lateral recumbency and, subsequently, in dorsal recumbency on a non-stationary MRI table. Dorsal and transversal slices of the heads were assessed. All CT scans were performed using a 16-line Brilliance™ CT—Big Bore Oncology Scanner (Philips Medical System, Best, The Netherlands). The technical settings were 140 kV, 300 mAs, 1.5 mm collimation, a pitch of 0.9 and a reconstruction matrix of 1024 × 1024. Transverse image series, created with multiplanar reconstruction (MPR), were orientated in alignment with the teeth depicted. The MRI were obtained using a Philips Achieva™ 3.0TX-Series® MRI. Surface coils (Philips SENSETM FlexM® and Philips SENSETM FlexL®) were positioned around the region of interest, between the rostral margin of the facial crest and the orbital region. The MRI scans acquired were: T1 weighted (T1w), T2 weighted (T2w), proton-density weighted (PDw) and PDw fat-suppressed spectral attenuated inversion recovery (PD SPAIR). An additional table file shows this in more detail (see Additional file 1).

### Images analyses

After image acquisition, CT and MRI slices from different planes of the cheek teeth and adjacent structures were chosen (Table 1). Three slices through each of the maxillary 08s and 09s were chosen in a dorsal and transversal orientation in the CT, T2w, PDw and PD SPAIR scans.

**Table 1:** Evaluated structures depicted in imaging techniques and image alignments

Structures evaluated	Imaging techniques (alignments)
Dental tissues	
Pulp	CT (MPR), PDw (dorsal), PD SPAIR (transverse), T2w (transverse, dorsal)
Common pulp chamber	CT (MPR), PDw (dorsal), T2w (dorsal)
Dental hard tissues (enamel, dentine and cementum), alveolar part	CT (MPR), PDw (dorsal), PD SPAIR (transverse), T2w (transverse, dorsal
Dental hard tissues (enamel, dentine and cementum), clinical crown	CT (MPR), PD SPAIR (transverse), T2w (transverse, dorsal)
Periodontal tissues	
Periodontal ligament	CT (MPR), PDw (dorsal), PD SPAIR (transverse), T2w (transverse, dorsal)
Lamina dura of the maxillary bone	CT (MPR), PDw (dorsal), PD SPAIR (transverse), T2w (transverse, dorsal)
Adjacent tissues	
Cortical bone of the maxillary sinus	CT (MPR), PDw (dorsal), PD SPAIR (transverse), T2w (transverse, dorsal)
Mucosa of the maxillary sinus	CT (MPR), PDw (dorsal), PD SPAIR (transverse), T2w (transverse, dorsal)
Bony infra-orbital canal	CT (MPR), PD SPAIR (transverse), T2w (transverse)
Soft tissue inside the infra-orbital canal	CT (MPR), PD SPAIR (transverse), T2w (transverse)

*CT* computed tomography, *MPR* multiplanar reconstruction, *PDw* proton-density weighted, *PD SPAIR* proton-density weighted spectral attenuated inversion recovery, *T2w* T2 weighted

Predefined anatomical landmarks were used to ensure comparability of the selected slices. The dorsally orientated slice in the mid-tooth section of the maxillary teeth, for example, was selected after each tooth’s half-length was determined in the transverse scans. Each structure visible in this slice was scored. Data were exported in DICOM format to easyIMAGE software (easyVet, IFS Informationssysteme GmbH, Langenhagen, Germany). Images were analysed and evaluated on a 19″ flat DICOM certified TFT display (EIZO FlexScan MX190S; EIZO Europe GmbH, Mönchengladbach, Germany).

The images chosen were evaluated independently by three experienced veterinarians (MH, an experienced radiologist and resident in the European College of Veterinary Diagnostic Imaging; ABZ, a board-certified specialist in equine dentistry and CR, a-trained veterinarian). The CT and MR images were graded separately, and information concerning the specimen’s condition were hidden. A modified four-point rating scale was used by each observer to analyse the image quality (Table 2), as described in several human and veterinary studies evaluating imaging techniques [15, 18, 19]. Additionally, the visibility and the differentiation (contours and tissue distinction) of specific dental, periodontal and adjacent structures were graded (Tables 1 and 3). The examiners could adjust the window width and level individually.

**Table 2:** Modified scoring system for image quality parameters, according to [18]

Score	Image noise	Image sharpness	Image contrast
0 Poor quality…	… with very high image noise level or image quality highly impaired due to image noise	… due to low image sharpness	… due to low image contrast
1 Moderate quality …	… with high image noise level or image quality impaired due to image noise	… due to moderate image sharpness	… due to moderate image contrast
2 Satisfactory quality …	… with moderate image noise level or image quality slightly impaired due to image noise	… due to satisfactory image sharpness	… due to satisfactory image contrast
3 Good quality …	… without image noise or image quality impaired due to image noise	… due to high image sharpness	… due to high image contrast

**Table 3:** Modified scoring system for the visibility/distinction of anatomical structures, according to [18]

Score	Criteria for visibility of anatomical structures: Structures …	Criteria for tissue or contour distinction (differentiation): Contours…/Tissues…
0	… not visible	… not distinguishable
1	… poorly visible, but detectable; identified by its location and signal intensity, not by margins, shape or size	… distinguishable, but often blurred
2	… clearly identified by its location, signal intensity and shape, margins not clearly delineated	… well distinguished and seldom blurred
3	… very well visualized and clearly delineated by location, shape, signal intensity/density, size and margins	… very well distinguished and sharply defined

### Statistical analysis

Data were collected on spreadsheets (Excel® 2010, Microsoft® Corporation Redmond, Washington, USA). SAS® software (SAS Institute, Cary, NC, USA) was used for the statistical analyses. GraphPad Software, Inc.® (La Jolla, CA, USA) was chosen for the graphical and statistical representations. Data were tested for normal distribution with Kolmogorov–Smirnov tests and analysed with a non-parametric statistical test (Friedman test). Wilcoxon matched pairs signed-rank tests were applied to calculate significant differences between CT and MRI scores. An adjusted α* was assessed using Bonferroni’s procedure to maintain study-related errors. Therefore, each individual hypothesis was tested at a significance level of α/m where αis the overall alpha level (0.05) desired and m is the number of hypotheses. The inter-observer agreement was analysed using McNemar-Boker tests and the Cohen’s kappa coefficient was calculated.

## Results

The CT images, PDw, PD SPAIR and T2w sequences were included in the study. The three-dimensional T1w scans were excluded because the quality was not good enough for further evaluation. The field of view in the MRI scans ranged from 180 to 250 mm in dorsally orientated sequences and from 160 to 220 mm in transversely orientated MRI for all groups. A total of 1080 images were evaluated, and 14,040 parameters were graded by all observers (8424 parameters in group A; 2808 parameters in each of group B and C).

### Image quality, structures’ visibility and MRI/CT differentiation in horses post-mortem (group A)

**Fig. 2:**
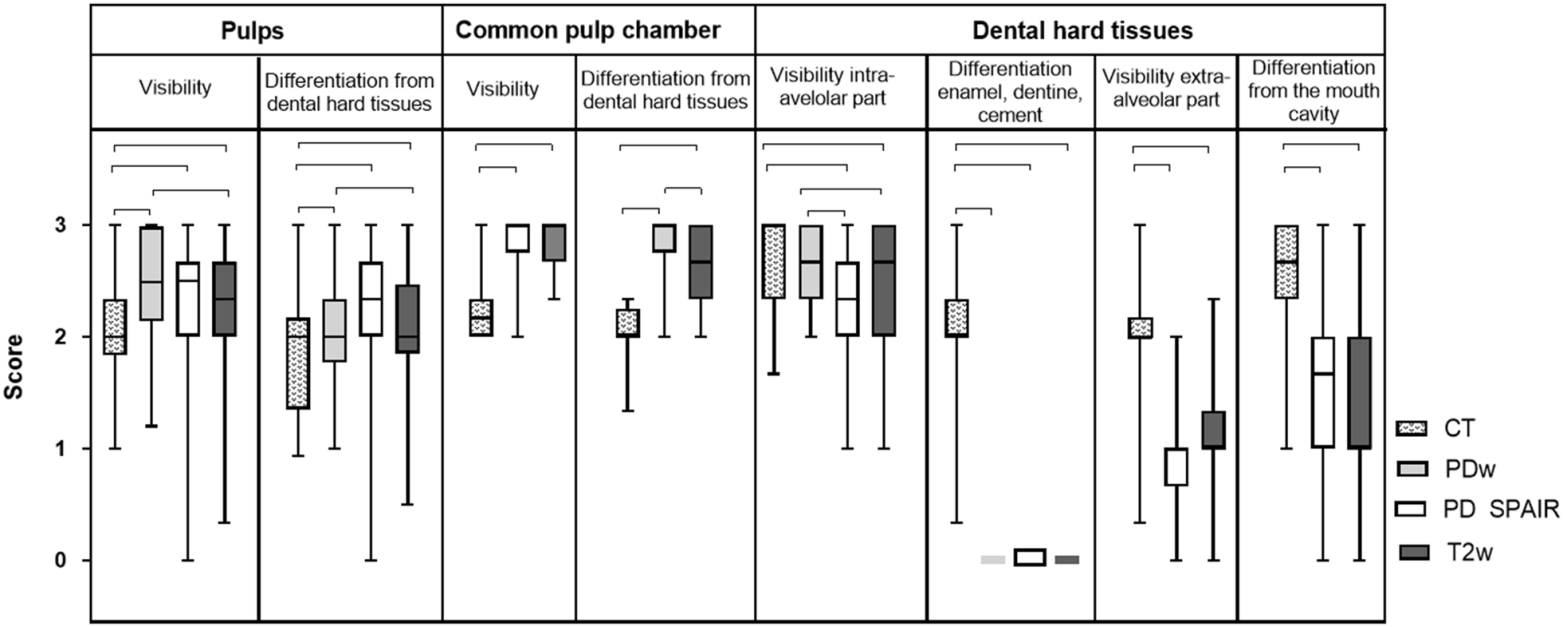
Graded visibility and differentiation of dental tissues comparing CT and MRI post-mortem (group A). Horizontal whiskers represent statistically significant differences between scores. Boxes represent the interquartile range and vertical whiskers the range. *CT* computed tomography, *PDw* proton-density weighted, *PD SPAIR* proton-density weighted spectral attenuated inversion recovery, *T2w* T2 weighted

**Fig. 3:**
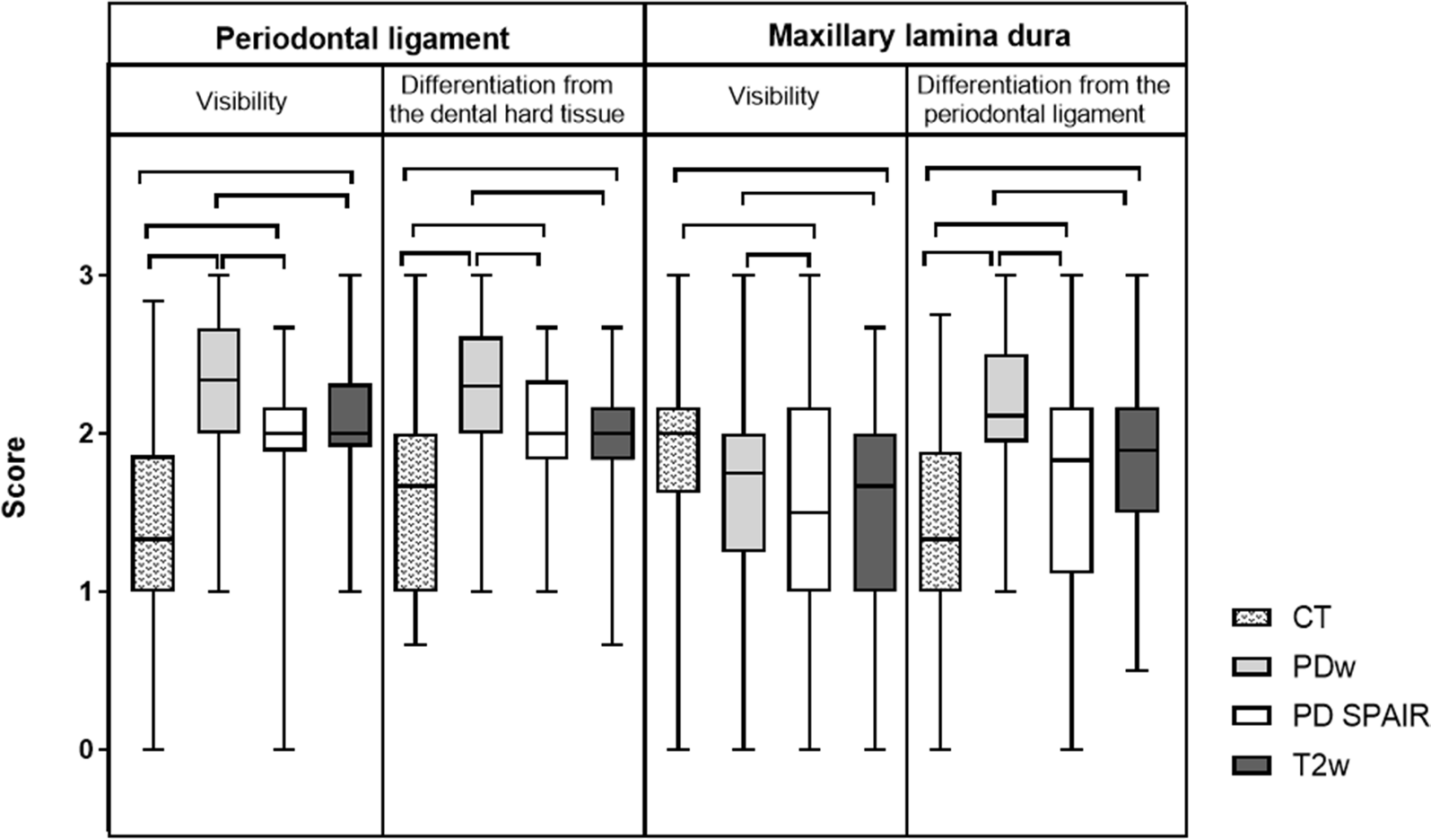
Graded visibility and differentiation of periodontal tissues comparing CT and MRI post-mortem (group A). Horizontal whiskers represent statistically significant differences between scores. Boxes represent the interquartile range and vertical whiskers the range. *CT* computed tomography, *PDw* proton-density weighted, *PD SPAIR* proton-density weighted spectral attenuated inversion recovery, *T2w* T2 weighted

**Fig. 4:**
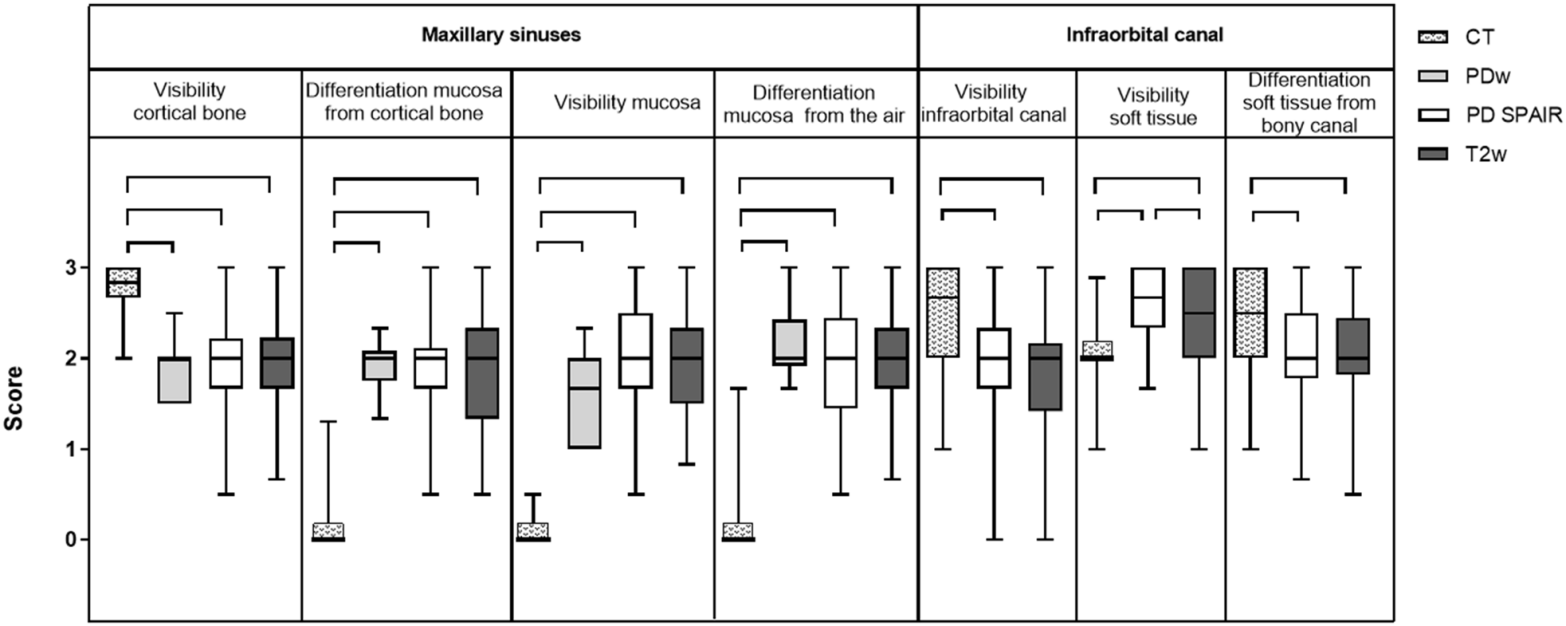
Graded visibility and differentiation of adjacent tissues comparing CT and MRI post-mortem (group A). Horizontal whiskers represent statistically significant differences between scores. Boxes represent the interquartile range and vertical whiskers the range. *CT* computed tomography, *PDw* proton-density weighted, *PD SPAIR* proton-density weighted spectral attenuated inversion recovery, *T2w* T2 weighted

**Fig. 5:**
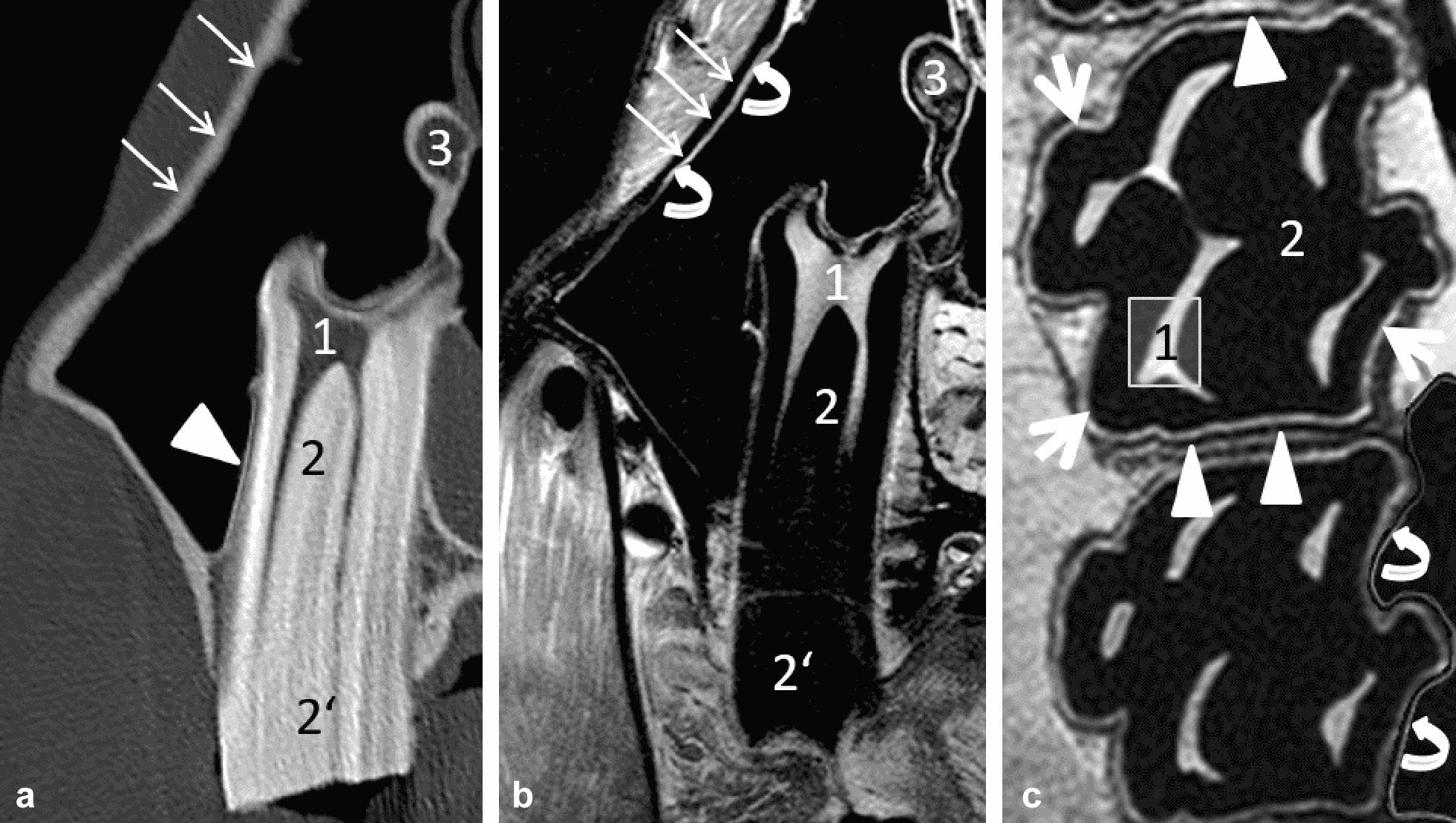
Dental, periodontal and adjacent structures evaluated. Transversal CT scan (**a**), T2w (**b**) and dorsal PDw (**c**) MRI sequences post-mortem (group A). 1 = pulp; 2 = intra-alveolar part of the dental hard tissues; 2′ = extra-alveolar part of the dental hard tissues; 3 = soft tissue inside the infra-orbital canal; thick arrows = periodontal ligament; arrowheads = cortical alveolar bone; thin arrows = cortical maxillary bone; curved arrows = sinus’ mucosa

Image quality parameters and scores for the visibility of dental (Fig. 2), periodontal (Fig. 3) and adjacent structures (Fig. 4) were analysed. The structures evaluated are depicted in Fig. 5.

The CT scores for image noise (median of 2.66) were significantly superior (P < 0.05) than the MRI scores for PDw (median of 2.13), PD SPAIR and T2w (median of 2.33) images (P (CT vs. PDw) = 0.0052; P (CT vs. PD SPAIR) = 0.0014; P (CT vs. T2w) < 0.0001). The CT scores for image sharpness (median of 2.66) showed significantly better results than the MRI scores for PDw, PD SPAIR and T2w images (medians of 2.33) (P (CT vs. PDw) = 0.0019; P (CT vs. PD SPAIR) = 0.0027; P (CT vs. T2w) = 0.0008). Image contrast was graded with a median score of 3 for all imaging techniques acquired and was not significantly different among all imaging techniques.

Regarding the structures’ visibility and differentiation from surrounding tissues, CT proved to be the superior imaging modality to display dental hard (enamel, cementum and dentine) and bony tissues (maxillary bone, infra-orbital canal): regarding dental structures, the visibility of dental hard tissues inside the maxillary bone (intra-alveolar part of the tooth), the differentiation of all dental hard tissues themselves, the visibility of the dental clinical crown and the delineation against the mouth cavity were rated higher in CT (P ≤ 0.001) compared to all MRI sequences (Fig. 2). Differentiation of the dental hard tissues and the oral cavity was only visible in MRI when hyperintense saliva or the tongue were next to the hypointense cheek teeth displayed, resulting in low median scores for PD SPAIR and T2w sequences (Fig. 2). Figures 3 and 4 show excellent visibility of the maxillary lamina dura, cortical bone and the infra-orbital canal on CT images.

In contrast to the CT images, 3.0 T MRI was the better imaging technique to display soft tissues. The visibility and delineation of pulp, the common pulp chamber, the PDL, the mucosa of the sinuses and the infra-orbital canal’s soft tissue achieved significantly better scores in MRI (P ≤ 0.0001) than CT. Nevertheless, the delineation of soft tissues against bony structures (e.g. the infra-orbital canal and the cortical bone of the sinuses) was only visible due to the hyperintense mucosa overlaying the hypointense outlining of the bone in MRI images. When comparing the MR sequences, the differentiation of dental soft tissues (pulps, common pulp chamber, PDL) from adjacent tissues was superior in PD SPAIR and significantly improved in PDw (P ≤ 0.001) sequences compared to T2w images (Figs. 2 and 3). Significant differences between the PDw and PD SPAIR sequence scores were evident for the periodontal apparatus: both the visibility (P < 0.0001) and the differentiation of the PDL from the dental hard tissues (P < 0.0001) and the maxillary lamina (P < 0.001) was significantly higher in PDw than PD SPAIR images (Figs. 3 and 4).

### Comparison of the image quality and defined visibility of the structures in vivo, post-mortem and frozen-thawed (group A, B and C)

Scores for the pulp, the PDL, the mucosa of the maxillary sinuses and the soft tissue of the infra-orbital canal were compared among group A, B and C and within the CT and the MRI. The PDw sequences of group B were compared with those of group A and C to compare the MRI scores among the different specimens’ conditions. The same applied to the PD SPAIR and T2w sequences.

#### Image quality scores

All the CT and MR images evaluated revealed good quality scores of > 2, including heads that had been frozen-thawed. Nevertheless, the image quality parameters differed between horses alive, post-mortem and frozen-thawed: image sharpness was rated significantly higher in CT (P ≤ 0.001, median score for group B = 2.32, group B = 2.66) and MRI (P ≤ 0.005, median score for group B = 2.13, group A = 2.33) in horses that were examined directly post-mortem than in live horses. Group C revealed median scores of 2.41 for CT and 2.24 for MRI without significant differences to the CT or MRI scores of group B (P = 0.16, P = 0.31) and A (P = 0.11, P = 0.23). Scores assessed for image noise did not differ significantly in CT imaging (P (A vs. B) = 0.53; P (A vs. C) = 0.40; P (B vs. C) = 0.28) or in MRI (P (A vs. B) = 0.37; P (A vs. C) = 0.21; P (B vs. C) = 0.30). Image contrast showed the best image quality scores, with values over 2.5 in CT (median score of 2.78 in group B, 2.72 in A and 2.65 in C) without significant differences among the groups (P (A vs. B) = 0.56; P (A vs. C) = 0.22; P (B vs. C) = 0.54). High-field MRI showed very good score values for image contrast in group B (median score of 2.8) and A (median score of 2.75). Both groups displayed superior image contrast scores compared to group C (median score of 2.61) but these differences were not significant (P (B vs. C) = 0.33; P (A vs. C) = 0.39).

#### Structures’ visibility scores

**Fig. 6:**
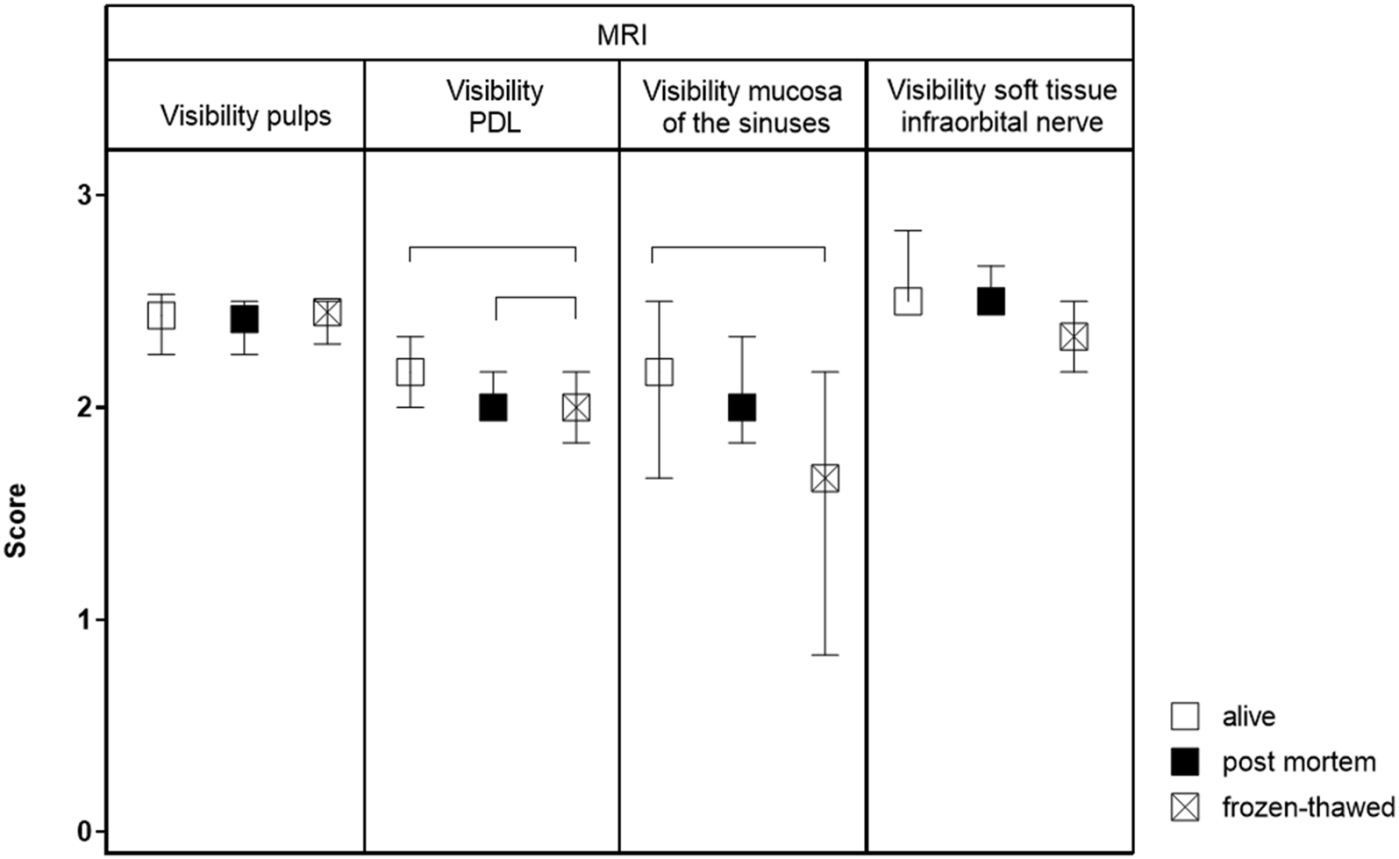
Graded MRI visibility in horses alive (group B), post-mortem (group A) and frozen-thawed (group C). Horizontal whiskers show statistically significant differences between scores. Boxes represent the interquartile range and vertical whiskers the range. *MRI* magnetic resonance imaging, *PDL* periodontal ligament

**Fig. 7:**
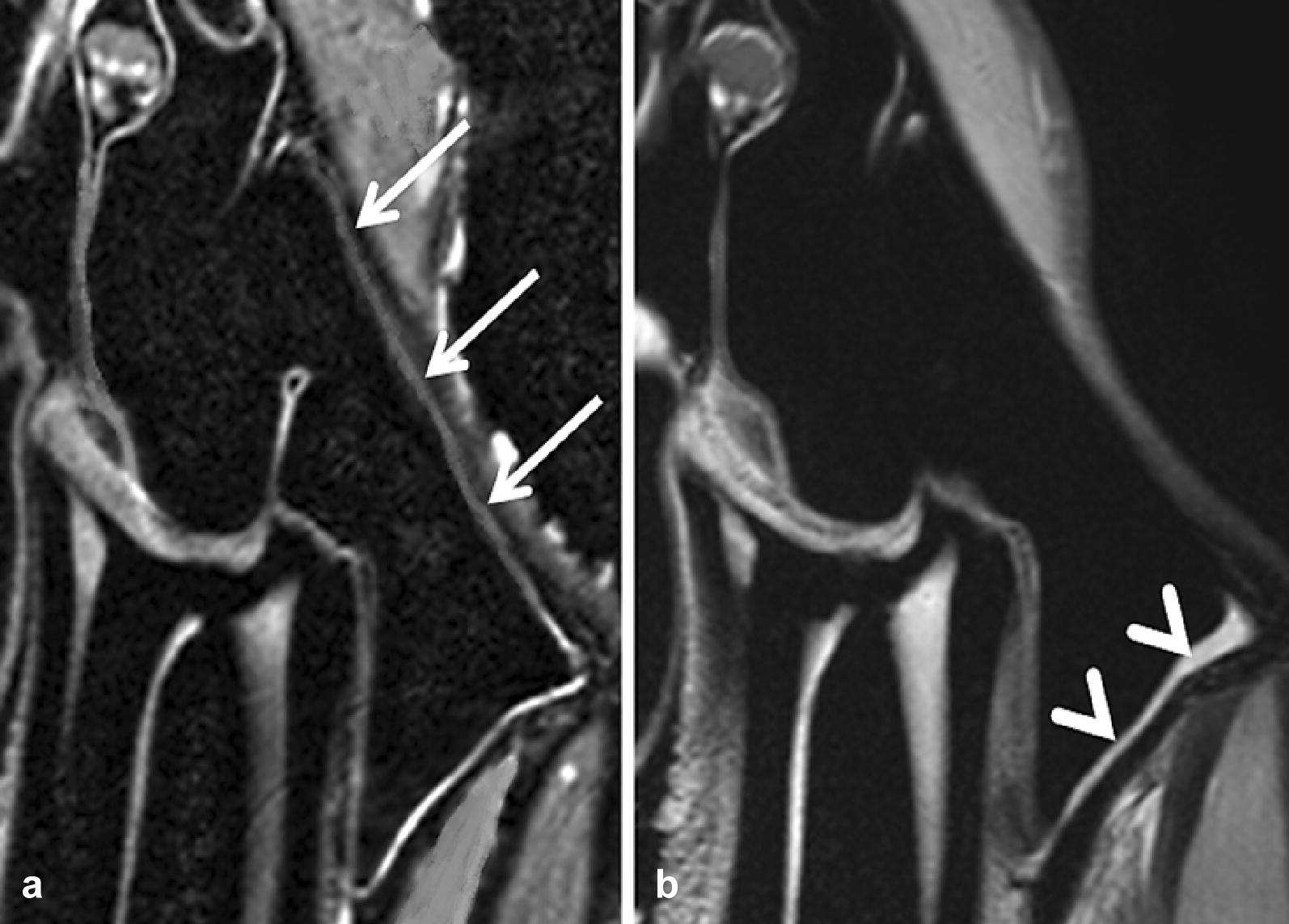
MRI findings in the same horse alive (**a**, group B) and post-mortem (**b**, group A). Both images display transversal T2w scans of a 209 (cheek tooth) and the periodontal structures. Arrows show the hyperintense sinus mucosa (**a**). Image noise is visible as hyperintense, cloudy signal within the hypointense sinus and the dental hard tissues (**a**) due to small patient’s movements. Arrowheads reveal thickened hyperintense mucosa (**b**) in the horse post-mortem

The CT scores for the visibility of pulp (P (A vs. B) = 0.12; P (A vs. C) = 0.46; P (B vs. C) = 0.79) and the soft tissue inside the infra-orbital canal (P (A vs. B) = 0.07; P (A vs. C) = 0.15; P (B vs. C) = 0.67) showed good score values in all groups without significant differences among the different groups and the MRI scores (Fig. 6) for the visibility of pulp (P (A vs. B) = 0.67; P (A vs. C) = 0.07; P (B vs. C) = 0.08) and the infra-orbital canal’s soft tissue (P (A vs. B) = 0.59; P (A vs. C) = 0.08; P (B vs. C) = 0.30). When comparing scores for the PDL, the CT scores did not differ significantly either (P (A vs. B) = 0.06; P (A vs. C) = 0.19; P (B vs. C) = 0.32). By contrast, the MRI showed significantly higher PDL score values in group B compared to group A (P = 0.006) or C (P = 0.001). Whereas the mucosa of the sinuses was not evident in CT scans of group A and B, some CT slices of heads frozen-thawed allowed the visualization of mucosa, resulting in higher score values. Nevertheless, the visibility scores of the mucosa were not significantly different between the groups in the CT scans. Regarding MRI, the mucosa had the best visualization in live horses (group B, Fig. 7a), significantly higher compared to group C (P ≤ 0.001).

### Inter-observer reliability

Calculation of inter-rater agreement for all raters showed good agreement in CT, PDw and PD SPAIR (P < 0.0001, Table 4), with a Kappa between 0.69 and 0.71. The inter-rater agreement for T2w was moderate between all raters with a Kappa of 0.59. Whereas the agreement between rater 2 and 3 and between observer 1 and 3 (despite T2w with good agreement) was very good in all imaging techniques, results for rater 1 and 2 just achieved good agreement. Rater 3 showed a tendency to evaluate all MRI sequences and CT scans with higher scores than the other two observers. Further evaluation of those tendencies showed that they did not affect the significance of the inter-modality comparison.

**Table 4:** Inter-rater agreement with weighted Kappa (wk) for each modality (P < 0.0001)

Modality		Raters compared			All raters
		Rater 1/2	Rater 1/3	Rater 2/3	
CT	wk	0.7750	0.8517	0.8695	0.7043
PDw	wk	0.7330	0.8135	0.8606	0.6921
PD SPAIR	wk	0.7495	0.8297	0.8841	0.7101
T2w	wk	0.6833	0.7921	0.8376	0.5953

*CT* computed tomography, *PDw* proton-density weighted, *PD SPAIR* proton-density weighted spectral attenuated inversion recovery, *T2w* T2 weighted

## Discussion

### Methodology

Recent studies described the qualities of CT [3, 9] or MRI [2, 20, 21] for the diagnosis of equine dental pathologies without any objective grading system to evaluate the different structures. The four-point rating scale used for the present examinations was designed to objectify results for a more detailed and less subjective comparison of CT and MRI. Whereas [18] focused on the CT and 3.0 T MRI’s ability to portray paranasal sinuses, to the best of the authors’ knowledge, the current study is the first evaluation which scores the quality of CT and MR images of equine dental tissues based on a grading system and compares the influence of the condition of the specimens on image quality.

### Imaging techniques/settings

Due to the technical progress, the CT examinations on standing, sedated horses provide a feasible alternative to CT under general anaesthesia [22, 23] used in the current study. The anaesthetic risk is reduced in sedated patients in comparison to general anaesthesia [24]. Nevertheless, scanning horses under sedation is not a simple procedure and requires a team of veterinary support staff. The main disadvantage of the standing procedure is movement blur, which may degrade the image quality or necessitate repetition of a scan. In the current study, authors tried to minimize the time of the scanning procedure to get the best image quality, but this might also have been achieved in images of standing CTs through repeated scans. In comparison to standing CT, where helical scanning is the only availability, axial scans with a longer scanning duration could be acquired under general anaesthesia and provide increased image quality. However, no standing CT was available in the clinic where the imaging acquisitions were performed. The total time of general anaesthesia could be shortened by using standing CT in further studies which combine CT and MRI examinations. This would result in a reduced anaesthetic risk in live horses and could permit longer MRI scan times if more image planes or MRI sequences are needed.

Comparing the examination times in all groups, the time needed differed markedly between CT and MRI: the CT was 13 times faster than the time taken for all the MRI scans. Whereas the MPR in CT scans provides the opportunity to create images in every alignment after the examination, a scan for each alignment is required in the MRI, resulting in long acquisition times. Finally, longer MRI examination times were chosen to produce high-quality images. Under clinical conditions, the number of MRI alignments or resolution might be decreased to reduce scan time and keep the anaesthetic time and risk as minimal as possible [25]. Three-dimensional T1w MRI scans offer the exception to produce MPR series. Nevertheless, T1w series were not evaluated further in the current study due to decreased image quality. High-field MRI requires long T1w scans, as the T1 relaxation time is prolonged [26]. In the present study, T1w image scans might have been too short to achieve satisfactory image quality and tissue visibility scores, therefore, the T1w three-dimensional sequence was excluded.

As a principal finding, all other MRI scans acquired in the current study proved to be capable of illustrating the regions of interest. Comparing different field strengths in MRI, the 3 T, caused by a twice as high signal-to-noise ratio compared to 1.5 T, allows for improved image quality and spatial resolution within the same examination time [27, 28].

### Cheek teeth selected

The maxillary 08s, 09s and 10s are the cheek teeth that show clinical signs, such as apical infections and infundibular caries, most frequently [1, 29]. As reported, the T2w, PD SPAIR and PDw scans evaluated did not acquire images in alignment with each tooth. Cheek teeth do not have the same alignments within one skull [30], so that the last upper teeth are not depicted mainly in perfect alignment [11]. Thus, selection was made for two adjacent cheek teeth with more similar angulations than the more caudally positioned upper cheek teeth to avoid low visibility and differentiation scores due only to the wrong alignment.

### Scores for CT, T2w, PDw and PD SPAIR sequences in group A (post-mortem)

Comparing the image quality in all CT and MRI scans evaluated of group A, the scores showed higher noise and less sharpness in the MR images. Reasons could be found in the MR coil positioning: whereas the whole head was scanned in the CT, examination coils were placed around the region of interest in the MRI, allowing a field of view of about 25 × 25 cm. The field of view in the current study ranged from 16 to 25 cm, therefore, mispositioning might lead to a decrease of signal intensity and image quality [18].

The authors of the current study agree with other investigations [11, 15, 31] that MRI is an ideal non-invasive technique to display soft tissue structures due to the increased water content of the latter. Consequently, MRI provided good to excellent visibility and differentiation scores for the soft and periodontal dental tissue detail and contrast, such as the pulp, PDL, mucosa of the sinuses, and infra-orbital nerve and vessels. A benefit of this ability to depict delicate soft tissues such as the infra-orbital nerve and its content, in clinical cases is that previously undetected pathologies may be visualized [31] even before they become visible with osseous changes in the CT.

As PDw and PD SPAIR sequences highlight tissues with a high proton density, superiority was found for both sequences compared to the T2w scans. Thin structures, such as the PDL, that is part of the periodontal apparatus, proved to be better visualized in PDw scans than PD SPAIR sequences. Tissues such as the PDL, that have a high free proton density, show a large transverse component of magnetization, depicted in a high signal [32]. In contrast to MRI, CT reached the lowest visibility score for the PDL of all structures that were displayed in the CT. Thus, MRI (especially PDw sequences) might be the more suitable imaging technique to prove whether the PDL is still vital. This could be used for presurgical planning in cases of endodontic procedures [12] or replantations [13, 14] in apically infected cheek teeth, as neither procedure is advised in cheek teeth with avital PDLs. Further investigations to evaluate the visibility of diseased PDLs in MRI are required.

Whereas CT scores good to excellent results, differentiation of the junctions and intra-oral air content of the dental hard tissues was poor in MRI, which is in line with the results in human medicine [19]. Unsatisfactory scores of bony and dental hard tissue structures are caused by the inability of conventional MR measuring methods to compensate for the very short relaxation times in hard tissues [33]. The MRI only provides an indirect depiction of structures with low proton densities: good visibility of the hypointense maxillary cortical bone and the infra-orbital canal were only possible because of their delineation against the hyperintense mucosa of the sinuses, and visibility of the extra-alveolar part of the dental hard tissues through the delineation against hyper- and isointense tongue tissue and saliva. These results suggest that CT is still the imaging technique of choice if osseous or dental structures are involved.

### Comparisons of scores between euthanized horses (A), live horses (B) and frozen-thawed cadaver heads (C)

Similar equine research studies described severe changes in MR image quality of soft tissues after freezing [16]. To prove, whether the image quality suffered in post-mortem or frozen-thawed soft tissues in the current study, the image quality and visibility of the pulp, PDL, mucosa of the sinuses and infra-orbital canal’s soft tissue were additionally evaluated in group B and C.

The results of the present study suggest that CT and MRI are excellent tools for good to excellent image qualities in all groups without significant differences in image noise and contrast. The image contrast was also satisfactory in heads that were frozen-thawed. The reasons could be that MRI does not measure signals for frozen materials in which atoms have lost mobility but yield a signal after the tissues thaw and reacquire molecular mobility [34]. These findings are in line with a previous study [16], where frozen limbs were defrosted and rescanned multiple times, resulting in no differences in the image quality of the scans. The high-resolution MRI presented and the CT examinations were susceptible to artefacts, resulting in worse image sharpness scores in group B than group A and C: small movements, through breathing and heartbeat, as was present in live horses, appeared as motion artefacts. The aim, therefore, should be to position and fixate the patient’s head properly and reduce the total measurement time in horses under general anaesthesia. Heads were fixed at the table in all groups in the current study; nevertheless, slight movement in live horses could not be prevented (Fig. 7).

**Fig. 8:**
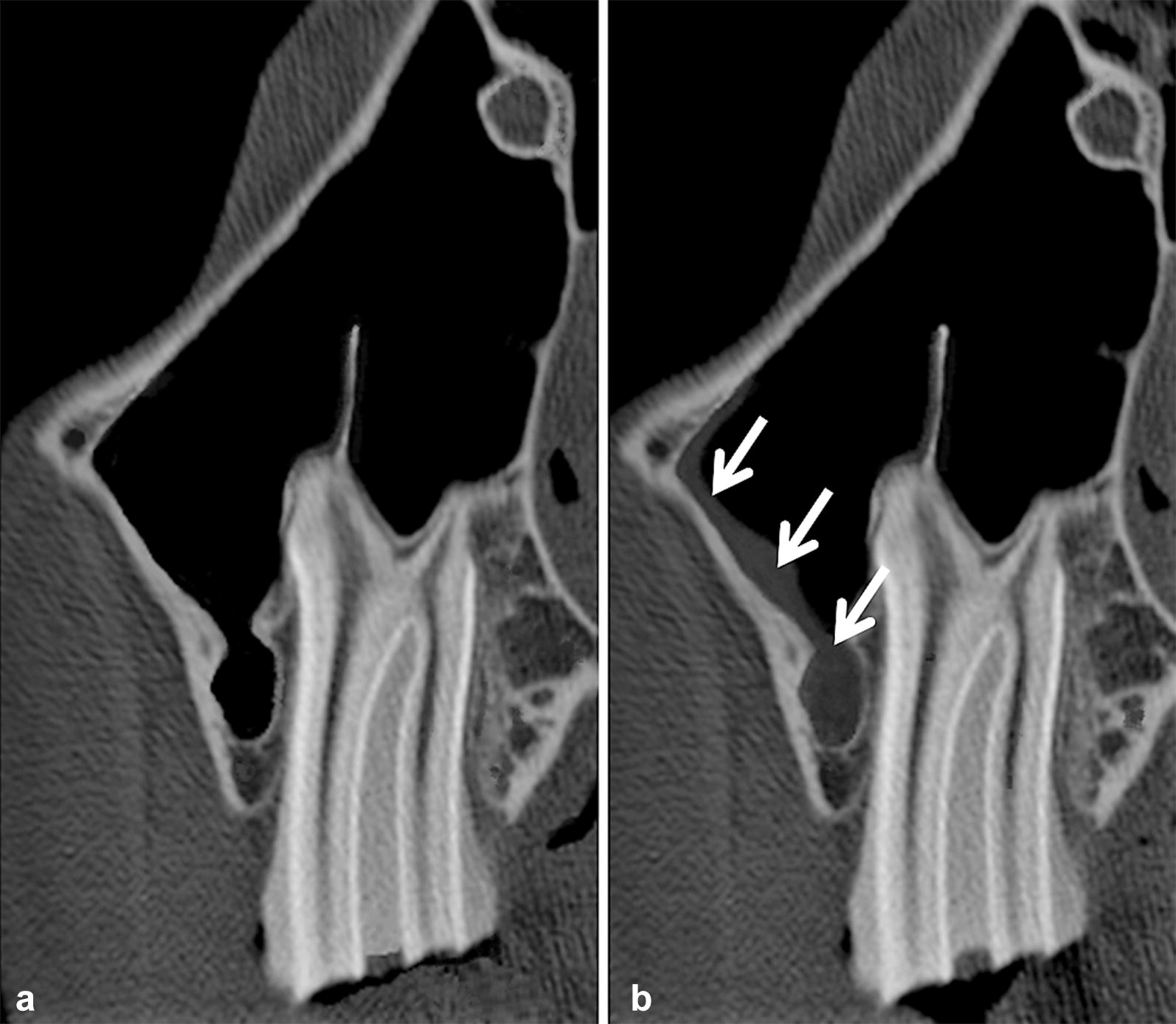
CT findings in the same horse alive (**a**, group B) and frozen-thawed (**b**, group C). Both images display transversal CT scans of a 109 (cheek tooth) and the periodontal structures. Arrows show the hyperdense thickened sinus mucosa in the head frozen-thawed (**b**)

The CT, depicting the hard dental and bony tissues, did not differ in any score of the soft tissues that were evaluated between group A, B and C, except for the mucosa of the sinus: mucosal oedema occurred during the freezing process, resulting in thickened mucosa that was visible in the CT scans in single heads (Fig. 8). Finally, regarding MRI research on paranasal sinuses, it must be considered that mucosa could appear pathological after freezing, although the horse had no thickened mucosa in vivo. This might lead to false positive results. A recent MRI study of frozen human vertebral columns described that freezing and thawing leads to a decrease of signal intensity for reasons such as incomplete core specimen thawing [35]. As the core temperature was checked for the specimens in group C, these artefacts should be prevented in the current study. It has been hypothesized that autolysis and water loss may contribute to changed, more hypointense MR signals after the freezing process [16]. These findings may explain the significant decrease in MRI visibility of the PDL and mucosa of the sinuses in group C. Other processes discussed with an MR signal decrease in tissues that had been frozen-thawed, such as meat, are protein denaturation and aggregation [36]. These mechanisms were accompanied by a reduction in T1 [36] and T2 [37] values. Although quantitative MRI of meat has shown that increasing the duration of the freezing period from 2 weeks to a month at − 18 °C does not significantly enhance protein denaturation [38], slight MR signal changes were visible in the current study. The significant superiority in visibility of the PDL in live horses compared to those post-mortem (group A) may be explained by the unavailable blood flow in euthanized horses.

In contrast to the PDL and the mucosa of the sinuses, no significant score differences were visible for the infra-orbital canal’s soft tissue and the pulp in the MRI scans. Both structures were surrounded by hard dental and bony tissue. Even if the cells’ integrity was destroyed in these structures, leading to a lower proton density, fluid cannot distribute in the oral cavity or the sinus. Thus, the surrounding structures may prevent a loss of MRI signal, depicting the extracellular fluid with a hyperintense signal.

Inter-rater agreement was good between all raters in the current examination and in a comparative study of MRI and CT regarding the equine fetlock joint [27].

### Clinical relevance

Scores of group A (n = 9) allowed for a comparison of CT, T2w, PDw and PD SPAIR sequences. Whereas CT highlighted mainly the dental hard tissues and bony structures, MRI provided a perfect depiction of soft tissues, especially in PD SPAIR and PDw sequences. In clinical cases, this knowledge might help to decide on an imaging technique or a specific MRI sequence in patients with dental disorders (e.g. PDw sequences to portray the PDL). With its good to excellent scores for bony and hard dental tissues, CT remains an auspicious method to portray pathological dental progresses concerning the alveolar bone (e.g. in cases of alveolitis) and structural abnormalities of the hard dental tissues (e.g. in cases of infundibular caries). Score results show that MRI might be a promising method to evaluate the vitality of the pulp and PDL in cases of endodontic treatment and replantation of infected cheek teeth. To the best of the authors’ knowledge, the correlation between MR signal intensity and the vitality of the PDL has not yet been verified in equine dentistry. Further studies with comparisons of the MR signal intensity and histological findings in diseased teeth are required to interpret the MR depiction of the PDL and pulp. In the end, the inherent problem that MRI examinations take up a long time if more sequences and orientations are necessary to evaluate pathologic processes must be considered.

### Limitations

Study limitations include that both live and euthanized horses, and the cadaver heads were removed from the CT and MRI gantry between imaging sections. The reasons for the changed signals might be due to a different placement of the coils and the head relating to the isocentre of the magnetic field. Moreover, a selection bias caused by the small size of the current study population cannot be ruled out. Image interpretation might not comply with the entire population of horses.

## Conclusion

The results of this experimental study suggest that CT is still the imaging technique of choice to portray bony structures and dental hard tissues. On the contrary, MRI provided a perfect depiction of soft tissues such as mucosa, the PDL and the pulpar tissue, especially in PD SPAIR and PDw sequences. The comparisons of the image quality between live, post-mortem and frozen-thawed specimens showed that image quality parameters did not suffer post-mortem or by freezing and thawing; the image sharpness was even better in these groups than in live horses and visibility scores were satisfactory for soft tissues in all specimen conditions. However, the authors’ hypothesis can be confirmed: significant superiority to portray the mucosa of the sinuses and the PDL was present in live horses. As such, the current study could serve as a reference for further research investigations to decide on the best specimen condition if a specific dental or periodontal structure is to be portrayed. In this context, recent MRI studies and results of horses with cheek teeth or sinus pathologies that were acquired after freezing must be regarded critically.

## Supplementary information

**Additional file 1.** Imaging techniques and parameters.

## Data Availability

The datasets used and/or analysed during the current study are available from the corresponding author on reasonable request.

## Abbreviations

CT: computed tomography
e.g.: exempli gratia = for example
MPR: multiplanar reconstruction
MR: magnetic resonance
MRI: magnetic resonance imaging
PDL: periodontal ligament
PD SPAIR: proton density weighted spectral attenuated inversion recovery
PDw: proton density weighted
T1w: t1 weighted
T2w: t2 weighted
TE: echo time
TR: repetition time
WL: window level
WW: window width
