# Supplementary figures and images for: Magnetic resonance imaging and computed tomography of equine cheek teeth and adjacent structures: comparative study of image quality in horses in vivo, post-mortem and frozen-thawed
Source: Acta Vet Scand. 2019 Dec 10;61:62. doi: 10.1186/s13028-019-0495-8 (PMC6905104; doi:10.1186/s13028-019-0495-8)

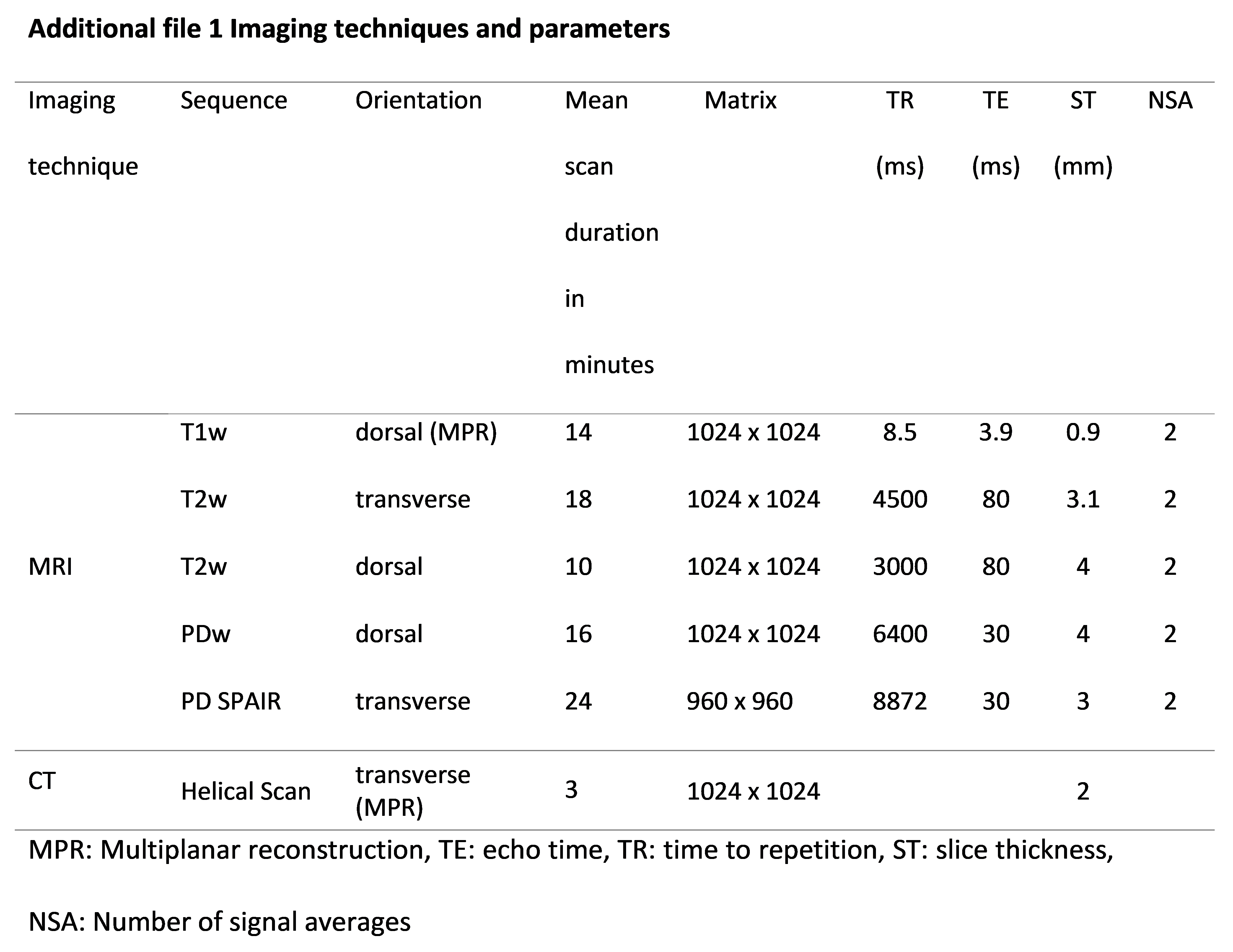

Supplement: Supplementary file 1 — Additional file 1. Imaging techniques and parameters. [file 13028_2019_495_MOESM1_ESM.png]
